# Data-driven hierarchical causal modeling of risk propagation in bridge operations: evidence from 132 accidents in China

**DOI:** 10.3389/fpubh.2025.1686346

**Published:** 2025-09-26

**Authors:** Peng Peng, Zuocai Wang, Peng Cui, Xiaokang Hu, Junfeng Yao, Sainan Lyu

**Affiliations:** ^1^School of Civil Engineering, Hefei University of Technology, Hefei, China; ^2^College of Civil Engineering, Anhui Jianzhu University, Hefei, China; ^3^School of Civil Engineering, Nanjing Forestry University, Nanjing, China; ^4^Department of Applied Physics and Electronics, Umea University, Umea, Sweden; ^5^School of Electrical Engineering and Automation, Hefei University of Technology, Hefei, China; ^6^School of Management, Hefei University of Technology, Hefei, China; ^7^School of Economics and Management, Anhui Jianzhu University, Hefei, China

**Keywords:** bridge operation safety, safety risk factors, risk propagation pathways, hierarchical risk modeling, operational hazard management

## Abstract

Aging bridges worldwide face growing safety challenges due to extended service life and environmental stressors. However, most existing studies lack a systemic perspective and mainly rely on fragmented, expert-driven assessments. Such approaches fail to capture the interplay of risk factors. This gap in understanding the interactions and propagation of risks limits the development of effective safety strategies for bridge operation. To address this gap, this study aims to identify and structure key risk factors affecting bridge safety in operational contexts by adopting a data-driven hierarchical model. Utilizing 132 officially documented accident reports from national safety databases in China (2007–2024), text mining techniques are applied to extract lexical risk items, which are subsequently refined through expert workshops and association rule mining to capture factor relationships. The Decision-Making Trial and Evaluation Laboratory (DEMATEL) method, integrated with Adversarial Interpretive Structural Modeling (AISM), is applied to construct a multi-level causal hierarchy of safety risks. The findings reveal 19 distinct risk factors, structured into seven levels with 20 transmission pathways. Notably, insufficient informatization management and unqualified managerial competence are identified as foundational factors, while overweight vehicle passage, inadequate inspection and maintenance, and geological and meteorological hazards emerge as direct triggers of safety incidents. The constructed hierarchy demonstrates a clear propagation chain from latent management deficiencies to observable surface-level hazards. Theoretically, the study advances the understanding of risk interaction mechanisms by integrating quantitative data analysis with expert interpretation. Practically, it provides infrastructure safety managers with a structured roadmap for targeted interventions, emphasizing the importance of enhancing digital management systems, traffic load regulation, and emergency preparedness in bridge operation contexts.

## 1 Introduction

The transportation system serves as a vital lifeline for national development and public welfare, with bridges constituting a critical component of the transportation network (1). However, as bridge infrastructure continues to advance and age, the safety risks associated with their operation have become increasingly significant. Globally, bridges face a range of challenges during operation and maintenance, including structural fatigue, functional degradation, insufficient load-bearing capacity, and a low level of digitalized management (2). China, possessing one of the largest bridge inventories in the world, is now witnessing a marked trend of aging in its in-service bridges. Over the past three decades, China has reported more than 300 incidents involving bridges in service, with up to 70% occurring during the operational phase (3). These events, including terrorist attacks, explosion threats, hazardous material transport accidents, overloading, and pier impacts, underscore the diverse hazards that can emerge after a bridge enters service. A recent commentary in *Nature* highlighted that the risks of bridge collapses during operation are real and expected to escalate, driven by climate change and the aging of bridge infrastructure (65). Such deterioration processes can exacerbate structural vulnerabilities, compelling engineers and policymakers to take decisive actions to ensure the safety of operational bridges. Ensuring safety throughout the operational lifespan has therefore become a global priority, as failures at this stage can lead to severe economic losses and far-reaching social consequences. With the global bridge inventory expanding rapidly, the urgency of addressing operational safety risks—particularly in China—has intensified. Risk assessment stands at the core of this endeavor, providing the foundation for preventive strategies that safeguard both infrastructure and public well-being.

Extensive research has been devoted to bridge safety management, with scholars examining risks from multiple dimensions including human factors, construction processes, and systemic interactions. Previous studies have highlighted that human error remains a critical contributor to bridge-related incidents, influencing decision-making, operational behaviors, and safety performance across different project stages (4, 5). At the same time, advances in modeling approaches—such as random Boolean networks, social network analysis, and other complex systems methods—have deepened understanding of the multifactorial coupling among human, equipment, management, and environmental risks, particularly during construction phases (6). While these findings have enriched the theoretical and methodological foundation for safety risk analysis, most attention to date has been directed toward the construction stage, leaving operational-phase risks comparatively underexplored despite their potentially greater consequences over the service life of bridges. Although safety risk assessment has been extensively studied in sectors such as coal mining (7), building construction (8, 73), subway construction (9) and metro system operations (10), the specific context of bridge operations remains significantly underrepresented in scholarly research. Moreover, the majority of existing studies have predominantly adopted knowledge-driven approaches—such as expert elicitation (9), structured questionnaires (11), case-based analysis (12), literature synthesis (13), and on-site investigations (14)—which, while valuable, often introduce subjectivity and lack consistency in factor identification. In contrast, data-driven methodologies that enable objective and scalable extraction of safety-related patterns from historical records have seen limited application in the bridge safety domain. Furthermore, although some recent studies have incorporated systems-thinking perspectives to analyze risk propagation (15–17), the existing literature remains fragmented and seldom addresses the complex, hierarchical, and nonlinear interrelations among diverse safety risk factors in operational bridge environments.

To address these theoretical and practical gaps, this study aims to systematically identify and structure the key safety risk factors affecting bridge operation by leveraging data-driven and hierarchical modeling approaches. Although numerous risk elements have been mentioned across prior studies, a coherent, multi-level structure capturing their causal interdependencies remains largely underexplored—especially during the operational lifespan of bridges. This study focuses specifically on risk identification and propagation in the bridge operation phase, which is often overlooked compared to construction-phase analysis. Accordingly, three specific research objectives (*RO*s) are proposed:

*RO*1: To identify and distill the key safety risk factors associated with bridge operations from large-scale accident records.

*RO*2: To reveal the interrelationships among these risk factors and determine the most critical and recurrent risk patterns.

*RO*3: To establish a hierarchical risk framework and map the pathways through which risks propagate during bridge operations.

To address these objectives, the study draws on 132 officially documented accident cases involving bridge operations in China between 2007 and 2024. Key operational safety risk factors were identified and distilled through computational text mining and refined via expert validation (for *RO*1). Their interrelationships and recurrent patterns were uncovered using association rule analysis (for *RO*2). Finally, an integrated DEMATEL–AISM modeling approach was employed to construct a hierarchical risk framework and map the pathways through which risks propagate during bridge operations (for *RO*2 and *RO*3). This multi-phase analytical design combines large-scale empirical evidence with structured causal modeling, ensuring both the robustness of the results and the clarity of the systemic insights obtained.

Theoretically, this research advances the field by integrating data-driven extraction techniques with hierarchical modeling to illuminate the multi-layered dynamics of operational bridge safety risks. It offers a novel lens to conceptualize risk propagation pathways and addresses prior gaps in fragmented or oversimplified risk identification frameworks. Practically, the findings inform infrastructure safety governance by providing a structured foundation for targeted interventions across different organizational levels—ranging from digital management system upgrades and inspection protocols to traffic control and emergency response mechanisms. The insights are particularly relevant for countries with aging bridge inventories and rapid urbanization, where operational risks are both complex and consequential.

## 2 Literature review

### 2.1 Bridge safety risk management

**Figure 1:**
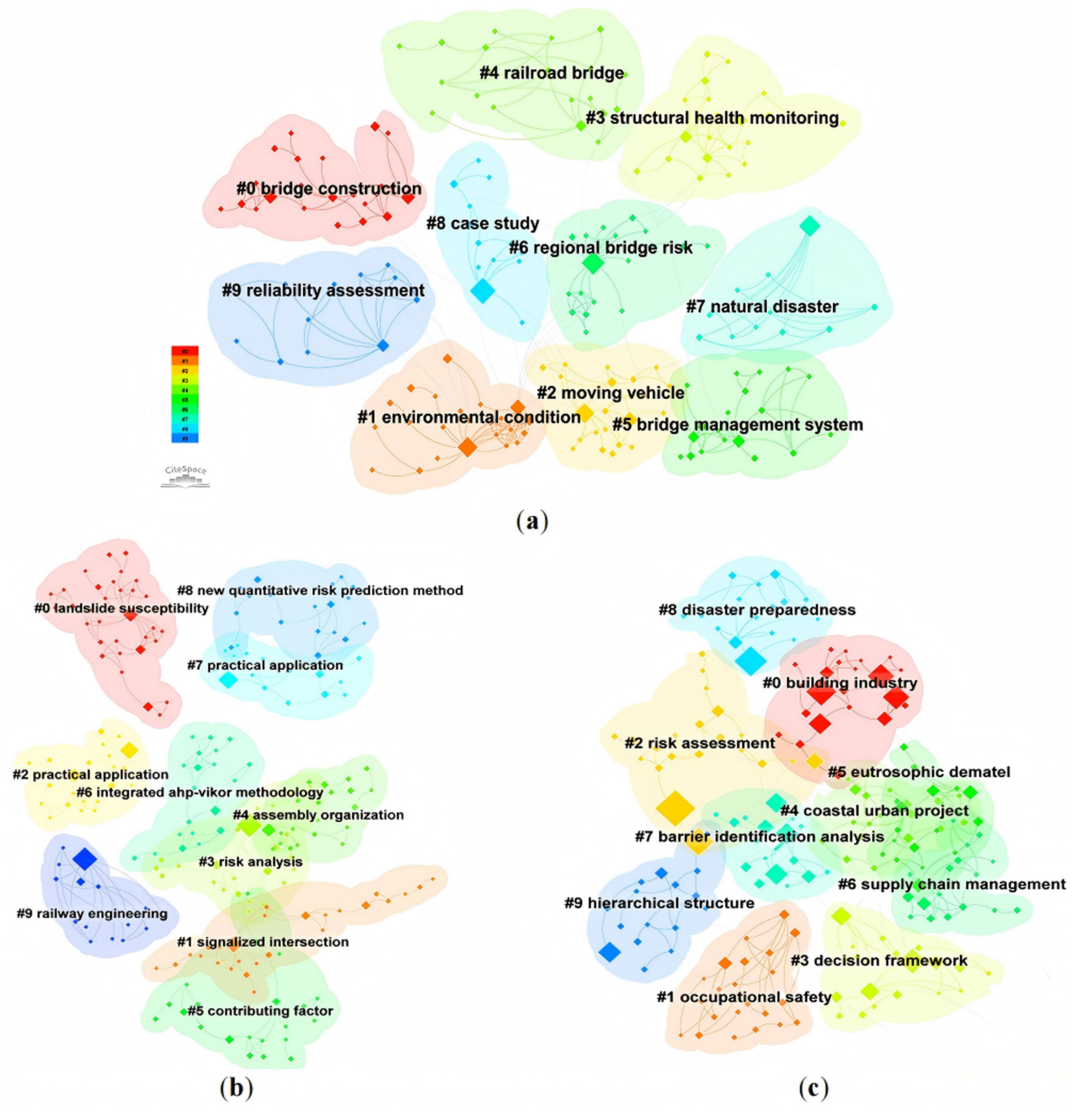
Cluster view of knowledge domains. **(a)** Bridge safety risk management. **(b)** Identification and evaluation of safety risk factors. **(c)** DEMATEL-AISM/ISM method.

Bibliometric analyses of recent publications on bridge safety risk management, evaluation of safety risk factors, and the application of DEMATEL-AISM/ISM methods are summarized in Figure 1. Among these, Figure 1a specifically illustrates the distribution of research topics in bridge safety risk management, showing that the majority of studies concentrate on the construction phase of bridges.

In the construction phase, the most frequently examined risk factors include environmental conditions, human errors, and natural disasters, while common methodological approaches involve structural health monitoring (SHM), bridge management systems (BMS), reliability assessment, and detailed case studies (1). These bibliometric findings align with earlier literature (18–20), which consistently reports that safety research during construction has received the bulk of scholarly attention. Representative examples include investigations into human error-induced risks in bridge construction (4), analyses of multi-factor coupling mechanisms in construction accidents (6), and studies linking worker behavioral traits to safety performance (5). Such works have contributed to a solid understanding of technical, human, and environmental risk interactions during construction, while advancing methodological innovations in safety assessment, such as reliability modeling, network analysis, and decision support frameworks.

In contrast, research on safety risk management during the operational phase of bridges remains comparatively limited, despite growing global concern over aging infrastructure, cumulative load effects, and climate change–induced hazards (1, 65). Existing operational-phase studies have largely focused on targeted technical interventions. For example, SHM systems have been applied to detect fatigue cracks, corrosion, and deformation in real time, supporting preventive maintenance (1, 21). BMS integrating inspection records, sensor data, and maintenance planning have been developed to optimize resource allocation and extend service life (19, 22). Accident case analyses have also been employed to identify recurring hazards in service, such as overloading, insufficient inspection frequency, and inadequate emergency preparedness (3, 23). Some works have begun to incorporate probabilistic risk models and resilience assessment frameworks to evaluate operational safety under extreme events, including earthquakes and floods (24, 25).

While these operational-phase studies have yielded valuable insights into specific risk control measures and monitoring technologies, their focus has generally remained on discrete aspects—such as structural health, load management, or incident response—rather than on the integrated management of complex, interacting risk factors across the operational lifecycle. Given the rapid expansion of bridge inventories worldwide, the aging of existing structures, and the intensifying impacts of climate change, advancing the understanding of safety risks in the operational phase is both urgent and globally relevant. Addressing this gap not only supports more resilient infrastructure systems in China but also contributes to the international body of knowledge on life-cycle safety management for critical transport assets.

### 2.2 Identification and evaluation methods of safety risk factors

Compared with the construction stage, systematic research on safety risk identification during the operational phase of bridges remains scarce. Existing studies in bridge safety risk identification and evaluation have relied on qualitative and expert-based assessments (2), typically incorporating elements of structural inspections and accident case analyses (23). While these approaches can provide rich domain-specific insights, they are limited in their ability to comprehensively capture complex and dynamic risk interactions in operational contexts.

To obtain a broader perspective on methodologies for safety risk identification and evaluation, a systematic review of relevant research was conducted. It is found that the identification and evaluation of engineering safety risk factors has evolved considerably over the past decades. Traditional risk factor identification methods were predominantly based on expert elicitation, structured questionnaires, and the analysis of historical accident reports (9, 26, 27). Such approaches, although rich in contextual understanding, often introduced subjectivity and inconsistency in the weighting of factors. In response, recent studies have introduced data-mining techniques, such as text mining (6), which provide a more objective basis for identifying influential parameters (28). The techniques help engineering operators to capture not only the common hazards (e.g., structural fatigue, overloading), but also the less conspicuous environmental and human factors that contribute to risk propagation (6, 29). However, the relevant implementation in bridge field is still rare. Apart from that, safety risk evaluation involves analyzing both the probability of hazardous events and their potential impacts (29). For instance, Xue et al. (73) investigated the safety risks of shield tunnel construction undercrossing rivers (STUR) by combining literature review, expert discussion, and a hybrid grey-DEMATEL-ISM approach, identifying 32 risks across four categories and elucidating their interrelations with potential impacts, which demonstrates the potential of integrated causal-hierarchical analysis frameworks for complex infrastructure projects.

Bibliometric clustering results in Figure 1b reveal that existing research on safety risk factor identification and evaluation is concentrated in several prominent thematic areas, including landslide (#0), railway engineering (#9, including bridges), new quantitative (#8), and integrated methods (#6). Recent relevant research on infrastructure project operations primarily focuses on identifying critical contributing safety risk factors in traffic management (66), subway operation (67), bridge construction (22), and coal mining (68) by developing tailored evaluation models and indicator frameworks.

In parallel with text mining and traditional statistical methods, recent studies in civil engineering have demonstrated the growing application of advanced machine learning (ML) and artificial intelligence (AI) models for safety risk identification and evaluation. For example, a cascaded deep learning framework has been developed for pavement crack detection and segmentation, enabling location-aware feature extraction from large-scale infrastructure images (30). Similarly, a physics-informed neural network has been applied to predict stratified ground consolidation based on excess pore water pressure monitoring data, demonstrating how domain knowledge can be embedded into AI models for improved interpretability (31). In addition, ensemble learning methods such as stacking have been successfully introduced to predict the pullout capacity of small ground anchors, illustrating the potential of ML-based hybrid models in geotechnical safety prediction (32). These advances highlight that data-driven and ML-enhanced approaches can significantly improve the accuracy and scalability of risk identification. However, they are predominantly designed for component-level predictions (e.g., cracks, soil consolidation, anchors), whereas systemic and hierarchical propagation of safety risks in bridge operations remains insufficiently addressed.

Existing studies on safety risk identification and evaluation can broadly be divided into three categories: (1) Knowledge-driven approaches. These include expert judgment, Delphi surveys, analytic hierarchy processes, and qualitative causal analysis (33, 70). Their main strength lies in incorporating rich domain expertise and contextual understanding, which is valuable when empirical data are limited. However, they are susceptible to subjectivity and may lack reproducibility. (2) Data-driven approaches. With the availability of large datasets, techniques such as text mining, statistical modeling, and machine learning have been increasingly applied to identify risk factors and predict failure patterns (30, 31, 34). Their strength is objectivity, scalability, and the ability to capture hidden patterns across large samples. Nevertheless, such methods may overlook tacit knowledge and require substantial computational resources and data quality assurance. (3) Hybrid or integrated approaches. Methods such as DEMATEL–ISM, Bayesian networks, and fuzzy logic models combine expert input with quantitative modeling to analyze complex causal relationships (2, 29). Their advantage is the ability to balance interpretability and analytical rigor, providing structured hierarchical frameworks and quantifiable causal pathways. The limitation is that model outcomes may still depend on the quality of expert input or parameter calibration. In summary, each approach has unique strengths: knowledge-driven methods emphasize expert insight, data-driven methods enhance objectivity and generalizability, and hybrid methods enable structured causal modeling. Building upon these, the present study integrates text mining, association rule mining, and DEMATEL–AISM to leverage the advantages of each while mitigating their limitations, thereby offering a more robust and systemic framework for analyzing risk propagation in bridge operations.

In the context of bridge operations, however, systematic identification and evaluation of operational safety risk factors remains limited. Most operational studies rely on expert judgment and routine inspection records, failing to apply advanced data-mining approaches. Addressing this methodological gap requires a systematic, data-informed framework capable of integrating large-scale operational accident data with advanced causal and hierarchical analysis techniques. Such an approach can move beyond isolated factor assessment toward a holistic understanding of interrelated risks in bridge operations, which is the focus of the present study. The proposed integrated methodology, designed to address this gap, is elaborated in detail in the subsequent methodology section.

### 2.3 Gaps in research

Despite significant progress, current bridge safety research is disproportionately weighted toward the construction phase, while the operational phase—when most bridges spend the majority of their lifespan—remains underexplored. Given the increasing number of in-service bridges and the rising frequency of operational hazards, risk management during the operational lifespan warrants more systematic investigation. This underrepresentation limits our ability to anticipate and mitigate the unique and evolving risks of in-service bridges. Furthermore, most prior research relies heavily on knowledge-driven approaches, such as expert elicitation and case-based analysis, to identify safety risk factors. Although such methods offer valuable insights, they are often limited by subjectivity and inconsistencies in factor definition. While some scholars have begun to analyze accident reports, few have employed robust, quantitative, data-driven techniques, such as text mining, to comprehensively extract risk factors from historical safety records.

Moreover, the identified factors are often presented in isolation, lacking integration into hierarchical frameworks that capture causal interdependencies and transmission pathways. Addressing these gaps is essential for developing a comprehensive and structured operational risk management strategy for bridges. As a foundational step in risk assessment, the identification of risk factors should aim to be both comprehensive and structured to support consistency and traceability in subsequent modeling. Yet, few studies have explored the layered, causal interdependencies among risk elements, especially within the context of bridge operations. Despite the availability of systems analysis tools, limited attention has been paid to constructing hierarchical risk architectures or modeling transmission pathways that reflect real-world risk propagation dynamics.

## 3 Methodology

### 3.1 Research framework

**Figure 2:**
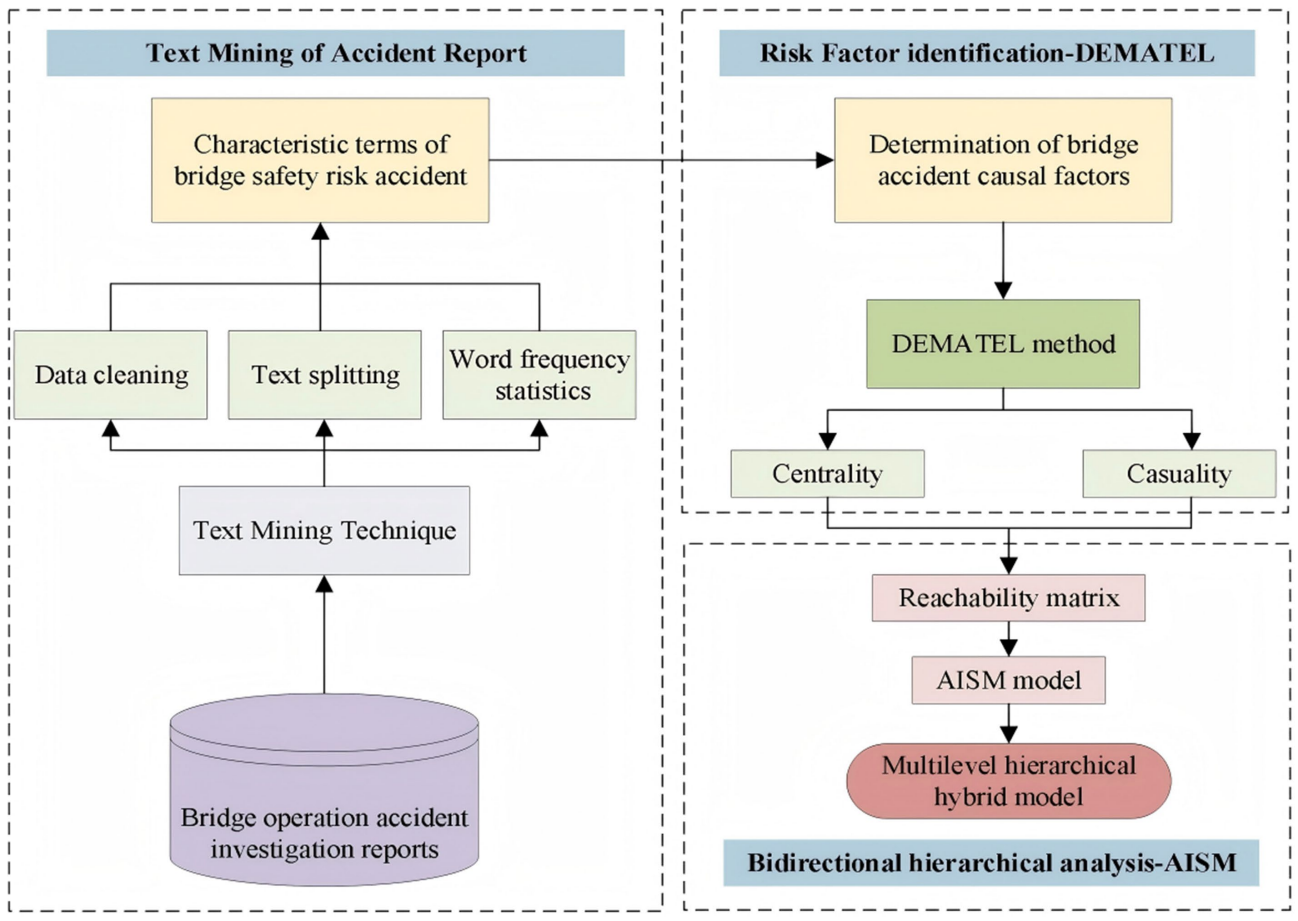
Research framework of the current study.

To systematically achieve *RO*1, *RO*2, and *RO*3, this study establishes a structured research framework (Figure 2) that sequentially identifies key safety risk factors, uncovers their interrelationships, and constructs a hierarchical risk framework to capture their transmission pathways during bridge operations. Initially, official accident investigation reports were retrieved from the National Ministry of Emergency Management (NMEM)^1^, the Safety Management Website^2^, and the Chinese Bridge Portal^3^. These reports were then refined through data cleaning to exclude irrelevant information and retain only content related to accident histories and underlying causes. Subsequently, text mining was employed to extract and summarize accident-related safety risk factors. Based on this, a dataset capturing the association patterns among these factors was constructed using association rule mining. Finally, a hybrid DEMATEL-AISM approach was applied to systematically examine the interrelationships and transmission mechanisms among the identified risk factors.

### 3.2 The integrated DEMATEL-AISM approach

#### 3.2.1 Decision-making trial and evaluation laboratory (DEMATEL)

DEMATEL is recognized as a powerful approach for uncovering causal relationships within complex systems (35). It facilitates the analysis of interdependencies among factors and identifies key elements through a visualized structural framework, which has been applied to many fields including systems engineering and management science. For instance, Agi et al. (36) employed DEMATEL to reveal interdependencies among 20 blockchain adoption enablers, highlighting technology advantage and external pressure as key drivers.

#### 3.2.2 Adversarial interpretive structure Modeling (AISM)

AISM method, built upon classical ISM and inspired by adversarial principles from Generative Adversarial Network (GAN), generates dual simplified hierarchical topologies through opposing extraction rules without compromising system integrity (37).

According to the bibliometric map as Figure 1c, DEMATEL, ISM and AISM methods have been widely used in the field of building industry, occupational safety, coastal urban project and supply chain management. DEMATEL and ISM/AISM methods are usually integrated used. To overcome the traditional DEMATEL method’s limitations, namely vague discrimination rules and subjective bias, this study applies AISM, which integrates inverse cause-based hierarchy extraction with ISM’s result-driven rules. This approach yields a clearer, more structured representation of complex interrelations through interpretable directed hierarchies (38).

Compared with the traditional ISM, AISM introduces a bidirectional adversarial extraction mechanism that generates both cause-oriented and result-oriented hierarchies. This dual perspective reduces the subjectivity inherent in single-directional ISM modeling, enhances structural interpretability, and allows clearer identification of dominant propagation pathways (29, 38). In this sense, the novelty of our approach lies in integrating DEMATEL’s quantitative causal intensity with AISM’s dual-hierarchy topology, which has not yet been systematically applied in the context of bridge operation risk analysis. Beyond the general reduction of subjectivity, AISM offers two specific advantages in the present study. First, by constructing both cause-oriented and result-oriented hierarchies, AISM enables verification from two perspectives, ensuring that the identified transmission chains of bridge operation risks are not artifacts of single-directional assumptions. Second, the dual-topology mechanism facilitates the detection of antagonistic or feedback relations that traditional ISM may obscure, which is particularly relevant given the intertwined nature of management, environmental, and technical risks in bridge operations (25, 29, 38). These advantages explain why AISM provides a more rigorous and transparent framework than conventional ISM for analyzing the propagation of operational safety risks.

#### 3.2.3 The combination of DEMATEL and AISM

Both DEMATEL and ISM are structural modeling methods that analyze interrelationships among factors through matrix operations and graph theory (25, 29). DEMATEL’s total influence matrix shares a high degree of similarity with the reachability matrix used in AISM, facilitating its derivation.

The integration of DEMATEL and ISM has been widely applied across various disciplines (29, 39, 40). As an enhanced extension of ISM, AISM incorporates a bidirectional adversarial hierarchy extraction mechanism, enabling clearer identification of structural differences and dominant transmission paths among factors. Thus, the combination of DEMATEL and AISM allows for a more precise depiction of causal propagation and structural contrasts within complex systems.

Recent studies have begun to adopt this hybrid DEMATEL-AISM framework and have reported promising results (38, 41). This integrated approach effectively captures the influence mechanisms, hierarchical layers, and transmission pathways of interrelated factors. In the context of bridge operation safety risk evaluation, DEMATEL identifies causal links, while AISM visualizes them through a bidirectional multi-level structure, thereby supporting the identification of key risk factors and offering a robust foundation for in-depth analysis.

### 3.3 Identification of safety risk factors

To achieve *RO*1, which is to identify and distill the key safety risk factors associated with bridge operations from large-scale accident records, manual extraction from numerous accident reports is time-consuming and prone to human error (42). Text mining, a data processing technique, enables the objective identification of valuable patterns from large-scale unstructured textual data (43, 44). Its effectiveness has been demonstrated across various domains. For instance, Shi et al. (29) identified 18 key causes from 127 coal mine construction accident reports; Zhou et al. (33) extracted 51 risk factors from 330 chemical accident reports and validated that text mining method can quickly and efficiently extract key information from incident reports; Das et al. (45) used text mining to extract key factors from 10 in-depth ambulance crash reports, revealing relationships of complex causes and enhancing public health safety.

Therefore, this study applies text mining to analyze bridge operation accident reports, following a workflow comprising data collection, preprocessing, structuring, analysis, and results output.

#### 3.3.1 Raw data collection

In 2007, the State Council of the People’s Republic of China issued the Notice on Conducting Safety Hazard Investigations of Major Infrastructure (69), mandating comprehensive safety inspections for critical infrastructure, including bridges, across the nation. This policy catalyzed the systematic collection of bridge safety data, resulting in standardized accident investigation reports maintained by authoritative bodies. To align with this policy and subsequent safety initiatives, this study leverages a robust dataset of 132 officially documented bridge operation accident reports from 2007 to 2024, covering a diverse range of bridge types, geographic regions, and incident severities. The dataset encompasses accidents involving various bridge categories, including beam, arch, cable-stayed, and suspension bridges, spanning urban, rural, and mountainous regions across China’s eastern, central, and western provinces. This diversity ensures broad representativeness, capturing a wide spectrum of operational contexts, from high-traffic urban lifelines to remote rural crossings. The reports, sourced from national safety databases, detail accident histories, causes, and contributing factors, providing a comprehensive foundation for identifying operational safety risks. The selection of the 2007–2024 timeframe aligns with the policy’s initiation and reflects a period of significant infrastructure expansion and aging in China.

To mitigate potential biases, such as overrepresentation of severe incidents or specific regions, the dataset was curated to include a balanced mix of minor, moderate, and catastrophic accidents, with incidents distributed across 28 provinces to reflect China’s geographic and infrastructural diversity. The use of standardized, government-verified reports enhances the dataset’s reliability and consistency, which offers a solid empirical basis for the text mining and hierarchical modeling approaches employed in this study and enables the systematic identification and analysis of safety risk factors in bridge operations.

#### 3.3.2 Data preprocessing

To ensure reproducibility of the text mining process, detailed preprocessing steps were documented, including removal of non-informative symbols, word segmentation, stop word filtering, and word frequency thresholding, as elaborated below.

Given the extensive length of accident investigation reports, only sections detailing the “accident history” and “cause analysis” were retained. Additionally, linguistic inconsistencies commonly found in such reports were corrected to ensure textual clarity and accuracy.

Furthermore, symbols and punctuation in accident investigation reports usually conveyed little semantic value for text mining, while unnecessarily inflating data dimensions and increasing the computational burden of training (46). To mitigate this, such elements were eliminated.

#### 3.3.3 Data structuring

Accident investigation reports, as unstructured textual data, should be transformed into machine-readable formats for effective processing. Since sentences convey meaning through word combinations, they require segmentation into individual words for further analysis.

Word segmentation refers to the algorithmic process of determining word boundaries within sentences or documents. It transforms lengthy passages into structured units of words, thereby facilitating subsequent processing and analysis. Unlike English, where spaces serve as natural delimiters, Chinese text lacks explicit separators (47). To address this, Jieba, a widely adopted segmentation tool, was employed to tokenize bridge accident investigation reports. However, while Jieba performs well with general vocabulary, it struggles with domain-specific terminology relevant to bridge operations. To improve segmentation accuracy, a customized dictionary was compiled to incorporate domain-specific terms absent from the default lexicon.

Stop words are high-frequency terms with limited semantic contribution, and thus are generally excluded in natural language processing. Their elimination reduces data volume and computational demands, which is particularly advantageous for resource-intensive algorithms such as Apriori, DEMATEL, and AISM, while still preserving meaningful content. In this study, the stop word list developed by the Harbin Institute of Technology (48) was adopted for filtering.

#### 3.3.4 Data analysis

In text mining, word frequency reflects how often specific terms appear, enabling macro-level visualization of key safety risk factors associated with accidents. Criteria for selecting word frequency thresholds were determined following prior studies (46, 49). Frequencies are listed in Table 1.

**Table 1:** List of safety risk factors in bridge operation.

Text mining word items	Factor refinement	Code	Frequency
Structure, span, superstructure, deck	High risk of bridge scale and structure	F1	85
Seismic zone, river, urban center, mountain	High risk of bridge location	F2	31
Collision, side-swipe, impact damage, vehicle	Frequent traffic collisions	F3	62
Fatigue, prolonged, material aging, wear and tear	Long bridge service life	F4	38
Technical grade, deterioration, steel grade, durability	Inadequate bridge technical grade	F5	25
Traffic, vehicle, congestion, trucks, lane	Complex traffic flow	F6	58
Remote sensing, monitoring, record, manual, management system, rules and regulations	Insufficient informatization management	F7	43
Expert, assessment, decision, advisory	Unestablished safety expert think tank	F8	23
Loading check, overload, traffic accident	Poor vehicle safety management	F9	62
Illegal sand mining, theft, arson	Deliberate human sabotage	F10	9
Overweight, overload, trucks, pass	Overweight vehicle passage	F11	98
Scour, earthquake, seismic zone, flood, landslide	Geological and meteorological hazards prone	F12	66
Hazardous cargo, chemical leakage, explosion	High risk dangerous goods transportation	F13	28
Arch bridge, beam bridge, historic bridge, cable-stayed	High risk of bridge type or culture	F14	43
Design deficiency, site planning, girders	Unreasonable planning and design	F15	65
Emergency response, unexpected, alarm, treatment	Unsound emergency management	F16	18
Hazard sources, chemical, fire, quarry	Nearby hazard sources	F17	44
Maintenance, inspection, repair, prolonged	Inadequate inspection and maintenance	F18	86
Timely, competence, management, recognition, experience, operation	Unqualified management competence	F19	35

#### 3.3.5 Result output

For text mining, PyCharm Edu 2024.1 was used as the compiler. The word items identified in the mining process are listed in the first column of Table 1.

#### 3.3.6 Expert-based refinement of risk factors

Drawing upon the text mining outcomes and accident report corpus, a structured expert workshop was conducted to refine the identified safety risk factors (50). Expert selection followed criteria derived from Hauashdh et al. (51), Luzon and El-Sayegh (52), Yusof et al. (53), and the approach of Shi et al. (29), requiring: (1) a minimum of a bachelor’s degree, (2) senior professional titles, and (3) at least 20 years of relevant experience in bridge engineering. Five experts meeting these standards were invited; details are presented in Table 2.

**Table 2:** Expert profile overview.

Expert	Age	Academic qualification	Professional title	Years of working
Expert 1	60	Bachelor	Senior engineer	35
Expert 2	57	Bachelor	Senior engineer	33
Expert 3	55	Master	Senior engineer	32
Expert 4	52	Master	Senior engineer	27
Expert 5	48	Doctor	Professor	21

The workshop proceeded in three phases: (1) Preparation phase. The workshop defined the research topic, expert group, and appointed a facilitator. (2) Discussion phase. Under the facilitator’s guidance, 4 iterative sessions were held as follows: Round 1: categorized lexical items into four domains: management, physical, environmental, and personnel; Round 2: further distilled these categories into 19 distinct risk factors; Round 3: aligned individual lexical terms with corresponding risk factors; Round 4: Reviewed and validated the full list of identified factors. (3) Synthesis Phase. The facilitator compiled the results and presented them for final expert review. No objections were raised, confirming the final risk factor set. And the frequency distribution of these refined factors is summarized in Table 1, with definitions detailed in Table 3.

**Table 3:** Connotation of safety risk factors of bridge operation.

Safety risk factors	Connotation
High risk of bridge scale and structure	Bridges with large spans or complex structural systems present high safety risk
High risk of bridge location	Bridges located in seismic zones, over major rivers, urban lifelines or mountainous terrain
Frequent traffic collisions	Vehicle or vessel impacts damage superstructure, triggering deck collapse hazards
Long bridge service life	Extended fatigue aging, wear cause material deterioration and sudden structural failure
Inadequate bridge technical grade	Poor bridge technical grade with low steel grade and poor durability
Complex traffic flow	High volumes, congestion and an unbalanced mix of multi-modal transport systems
Insufficient informatization management	Lack of sensor monitoring, BIM and digital management
Unestablished safety expert think tank	Missing expert assessment and advisory on safety risk
Poor vehicle safety management	Lack of overload control or route planning
Deliberate human sabotage	Intentional vandalism, theft, or arson
High risk of overweight vehicle passage	Overloaded trucks impose extreme deck stresses
Geological and meteorological hazards risk	Various hazards including scour, seismic shocks, floods, sandstorms or landslides
High risk dangerous goods transportation	Hazardous cargo leaks or explosions ignite fire on the bridge
High risk of bridge type or culture	Arch, cable-stayed or historic bridges with high safety risk
Unreasonable planning and design	Design defects and poor site planning with inadequate adaptation to evolving requirements
Unsound emergency management	Incomplete emergency plans and alarms delay response
Nearby hazard sources	Adjacent chemical plants or pipelines risk explosion
Inadequate inspection and maintenance	Inadequate inspections and deferred repairs
Unqualified management competence	Lack of professional expertise and operational experience

### 3.4 Analysis of safety risk factors through association rules

To address *RO*2, which aims to reveal the interrelationships among these risk factors and determine the most critical and recurrent risk patterns, the association rule mining method was employed to uncover underlying correlations among itemsets within the dataset (29, 54). Each safety accident case was examined to convert narrative descriptions into Boolean-structured data through enumeration. A value of “1” indicates the presence of a specific safety risk factor in a given case, while “0” denotes its absence. This process resulted in a structured dataset comprising 132 bridge operation accident cases, as presented in Table 4. In this table, each row (Tj) represents a unique accident case, and each column (Fi) corresponds to a distinct risk factor.

**Table 4:** Boolean dataset for safety risk association analysis in bridge operation.

Code	F1	F2	F3	F4	F5	F6	F7	…	F18	F19
T001	1	1	1	0	1	0	0	…	0	1
T002	0	0	0	0	1	0	1	…	1	0
T003	0	0	0	1	0	0	1	…	0	0
T004	1	0	0	1	1	0	0	…	1	0
…	…	…	…	…	…	…	…	…	…	…
T132	1	0	1	0	0	1	1	…	1	0

The Apriori algorithm, a foundational method for discovering association rules, was employed to investigate interdependencies among safety risk factors. Utilizing its rule-mining module, potential relationships between factor pairs were extracted. In alignment with previous research (29, 55), the threshold criteria were set as follows: Support > 0.001, Confidence ≥ 0.8, and Lift > 1. As a result, 32 meaningful rules were identified and are detailed in Table 5. Within the table, support indicates the relative frequency of occurrence, confidence reflects the predictive strength between items, and lift illustrates the nature and magnitude of their association.

**Table 5:** Safety risk factor association rules in bridge operation.

No.	Post-item	Pre-item	Percentage of support	Percentage of confidence	Degree of effect
1	F11	F9	35.67	93.45	2.5678
2	F9	F2	55.23	93.45	3.1234
3	F19	F7	32.12	92.34	1.7854
4	F18	F9	45.67	92.34	2.3456
5	F6	F3	40.12	91.78	2.2345
6	F11	F4	50.23	91.34	2.7654
7	F4	F15	45.67	91.23	2.3456
8	F18	F6	38.12	90.78	2.1234
9	F4	F1	38.90	90.56	2.1234
10	F11	F15	48.90	90.56	2.5678
11	F9	F6	36.78	90.12	1.8901
12	F12	F15	44.34	89.67	2.3456
13	F17	F15	30.12	89.45	1.8765
14	F16	F6	34.56	89.45	1.7890
15	F3	F6	37.89	89.34	1.7654
16	F5	F4	42.34	88.90	1.9876
17	F15	F19	25.34	88.76	1.5678
18	F6	F13	32.10	88.76	1.6789
19	F12	F16	30.12	88.45	1.8765
20	F5	F3	33.45	87.65	1.6789
21	F18	F19	25.34	87.65	1.6789
22	F18	F8	28.90	87.56	1.5678
23	F13	F6	30.45	87.23	1.5678
24	F9	F14	26.78	86.78	1.4567
25	F5	F13	28.90	86.54	1.4567
26	F16	F14	24.56	85.67	1.3456
27	F13	F5	27.65	85.34	1.3456
28	F10	F6	22.34	84.56	1.2345
29	F10	F14	20.12	84.34	1.1234
30	F3	F5	15.67	83.45	1.1234
31	F10	F2	12.34	82.34	1.0123

### 3.5 Analysis of safety risk factors through DEMATEL-AISM

**Figure 3:**
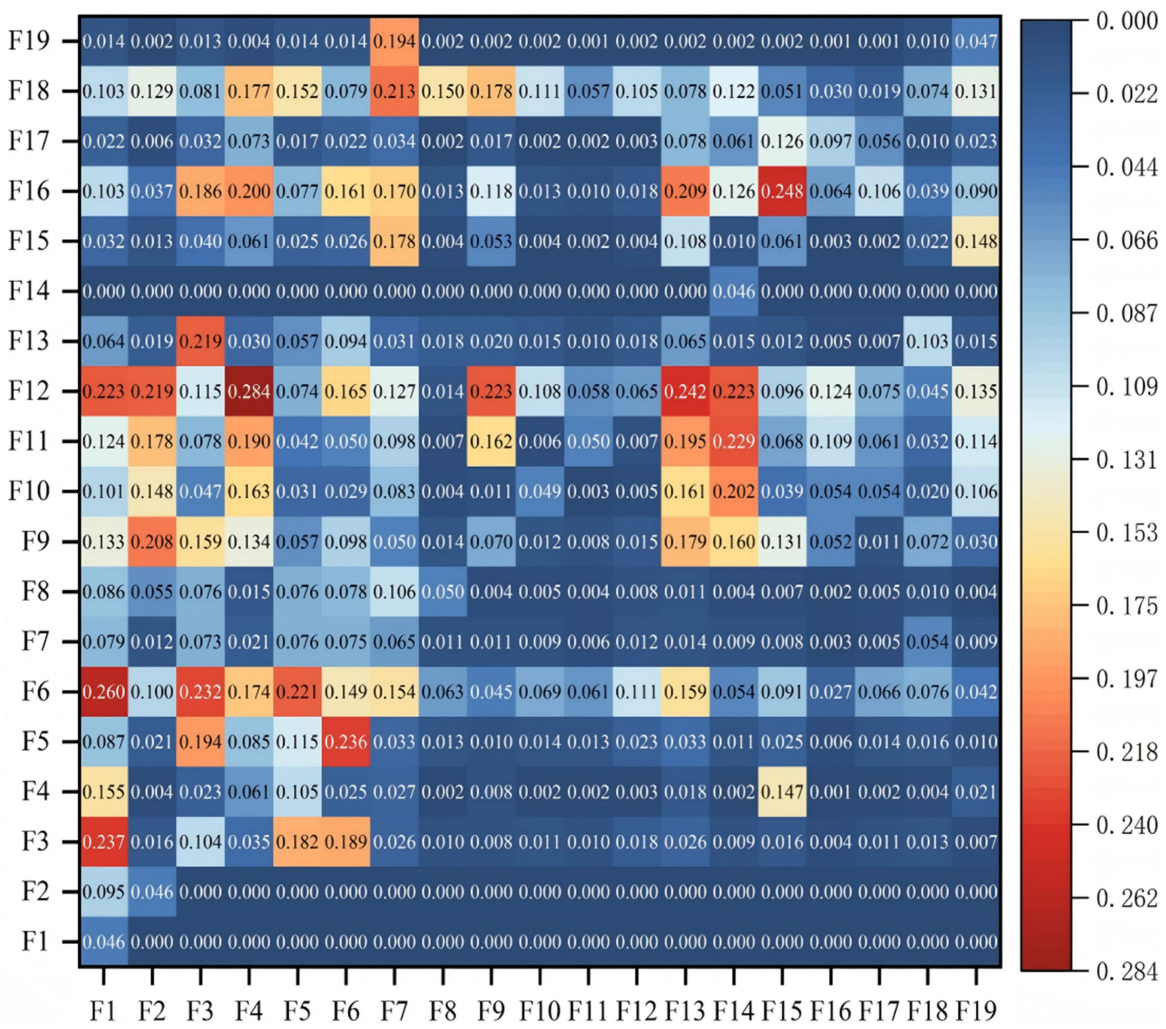
Total influence matrix.

To fulfill *RO*3, which seeks to establish a hierarchical risk framework and map the pathways through which risks propagate during bridge operations, the confidence values derived from the association rules were first used to construct a direct influence matrix, following the approach of Aaldering et al. (56). This matrix was subsequently normalized to generate a comprehensive influence matrix. By incorporating the identity matrix, a total influence matrix was formed, as presented in Figure 3. In this matrix, any element exceeding zero was encoded as “1,” and all others were set to “0.”

**Figure 4:**
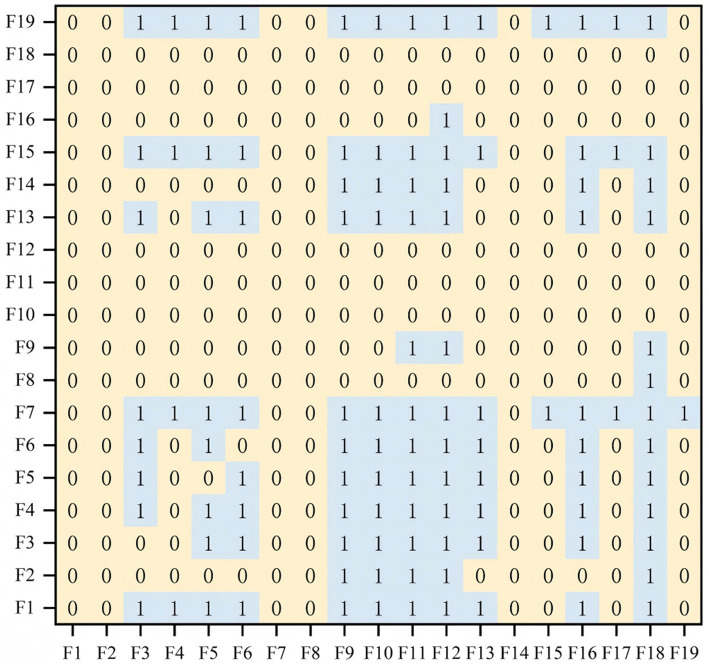
Reachability matrix.

To derive the reachability matrix (Figure 4), a transformation was performed based on a threshold *λ*. Given that the total influence matrix contains continuous values between 0 and 1, while the reachability matrix requires binary entries, elements equal to or above λ were assigned a value of “1”; otherwise, “0.”

Following previous DEMATEL-ISM applications, λ was determined by referencing the statistical distribution of the total influence matrix values. Specifically, elements were normalized within [0,1], and the mean value *μ* was adopted as the cut-off. This approach is widely used to balance network sparsity and connectivity, ensuring that only relations with above-average influence strength are retained while avoiding arbitrary subjectivity (25, 29, 35). To confirm robustness, a sensitivity test was also conducted by varying λ within μ ± *σ*/2, which showed consistent hierarchical structures.

## 4 Results

### 4.1 Identification of key safety risk factors in bridge operations

To address *RO*1, a comprehensive analysis was conducted on 132 officially documented bridge operation accident reports in China, spanning the period from 2007 to 2024. Through a text mining workflow involving data preprocessing, segmentation, and frequency analysis, an initial set of lexical items associated with accident causation was extracted. These preliminary results were subsequently refined through a structured expert workshop involving five senior bridge engineering specialists, each with over 20 years of professional experience. The iterative refinement process involved categorizing the lexical items into four thematic domains—management, physical, environmental, and personnel—and consolidating semantically similar terms to ensure conceptual clarity and eliminate redundancy.

The outcome of this dual-phase process was the identification of 19 distinct safety risk factors that comprehensively represent the operational safety risk landscape for bridges. These factors encompass a spectrum of hazards, from structural and environmental threats (e.g., geological and meteorological hazards prone, long bridge service life) to managerial and procedural deficiencies (e.g., insufficient informatization management, unqualified management competence, unsound emergency management), as well as traffic- and user-related risks (e.g., overweight vehicle passage, poor vehicle safety management, frequent traffic collisions).

The frequency distribution of these risk factors, summarized in Table 1, provides empirical evidence of their relative prevalence in real-world incidents, with overweight vehicle passage emerging as the most frequently cited cause, followed by inadequate inspection and maintenance. This structured and validated factor set forms the empirical foundation for subsequent analyses under *RO*2 and *RO*3, enabling systematic exploration of interrelationships and hierarchical propagation pathways within the bridge operation safety risk system.

### 4.2 Interrelationships and critical risk patterns from DEMATEL analysis

The influence degree measures how strongly a factor impacts other factors, while the affected degree reflects how much it is influenced by others. The centrality degree captures the extent of a factor’s connectivity with the rest, and the causality degree quantifies its role as a causal driver. The computed values for each safety risk factor across these four dimensions are presented in Table 6.

**Table 6:** Causal weight analysis of safety determinants via DEMATEL-based assessment.

Code	Influence degree	Affected degree	Centrality degree	Causation degree	Factor Properties
F1	1.962	0.045	2.007	1.917	Casual
F2	1.212	0.140	1.352	1.071	Casual
F3	1.670	0.932	2.602	0.738	Casual
F4	1.709	0.610	2.319	1.098	Casual
F5	1.321	0.955	2.276	0.366	Casual
F6	1.490	2.152	3.642	−0.663	Result
F7	1.588	0.551	2.139	1.037	Casual
F8	0.377	0.606	0.982	−0.229	Result
F9	0.938	1.593	2.531	−0.655	Result
F10	0.433	1.309	1.742	−0.877	Result
F11	0.296	1.799	2.094	−1.503	Result
F12	0.417	2.613	3.031	−2.196	Result
F13	1.578	0.816	2.394	0.762	Casual
F14	1.283	0.045	1.329	1.238	Casual
F15	1.127	0.794	1.921	0.333	Casual
F16	0.581	1.986	2.567	−1.405	Result
F17	0.491	0.683	1.174	−0.193	Result
F18	0.599	2.039	2.638	−1.441	Result
F19	0.930	0.327	1.257	0.602	Casual

Specifically, the influence degree characterizes a factor’s outgoing impact within the system, whereas the affected degree describes its susceptibility to external influences. Centrality degree represents the relational closeness of a factor to others, and causality degree identifies whether it primarily acts as a cause or consequence. As shown in Table 6, for example, overweight vehicle passage (F11) exhibits a low influence degree but a high affected degree, suggesting it is easily impacted by other risks and serves as a direct yet reactive contributor to incidents. It also means that while heavy vehicle passes are easy to recognize, they are inherently difficult to eradicate.

Conversely, for instance, insufficient informatization management (F7) scores high on influence degree but low on affected degree, indicating that it exerts widespread impact on other risks but is rarely influenced itself. This makes it a foundational driver in shaping safety risk dynamics in bridge operations.

Further analysis of centrality and causality degrees reveals that factors such as high risk of bridge scale and structure, high risk of bridge location, frequent traffic collisions, long bridge service life, inadequate bridge technical grade, insufficient informatization management, high risk of dangerous goods transportation, high risk of bridge type or culture, unreasonable planning and design, and unqualified management competence function as independent drivers, capable of triggering downstream risks. In contrast, factors including complex traffic flow, unestablished safety expert think tank, poor vehicle safety management, deliberate human sabotage, overweight vehicle passage, geological and meteorological hazards prone, unsound emergency management, nearby hazard sources, and inadequate inspection and maintenance, often manifest as consequences within the risk network.

These findings fulfill *RO*2 by revealing both the direct and indirect interdependencies among the 19 identified risk factors, highlighting the most critical drivers and recurrent patterns within the bridge operation safety risk network.

### 4.3 Hierarchical framework and propagation Pathways from AISM analysis

**Figure 5:**
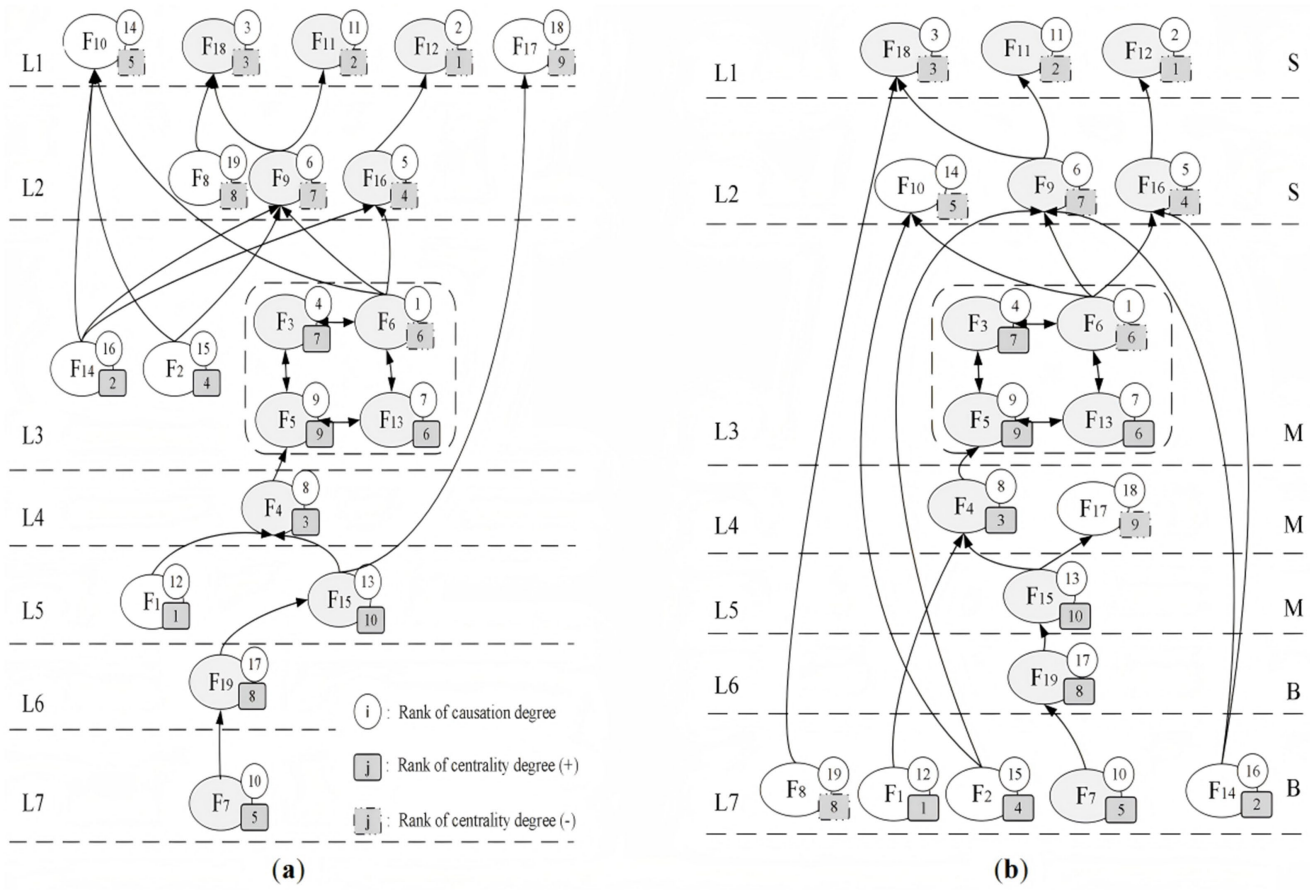
Hierarchical structure of safety risk factors. **(a)** cause-oriented hierarchical topology diagram **(b)** result-oriented hierarchy topology diagram.

In the reachability matrix, a value of “1” signifies that the horizontal node exerts a direct influence on the corresponding vertical node, whereas “0” indicates the absence of such a relationship. Based on this matrix, a directed hierarchical topology diagram was developed by mapping the relationships and antagonistic hierarchies identified among the elements (Figure 5). Specifically, Figure 5a depicts the cause-oriented hierarchical topology, whereas Figure 5b displays the result-oriented hierarchical structure.

Drawing on the AISM methodology, safety risks associated with overall bridge operation were classified into three hierarchical levels: superficial, intermediate, and fundamental. Superficial factors represent the most immediate and observable risks at the downstream end of the transmission chain, often directly limiting operational safety. Intermediate factors serve as transitional elements, indirectly contributing to risk development and functioning as a bridge between root causes and surface-level manifestations. And fundamental factors reside at the upstream origin of the risk transmission pathway and constitute the underlying causes impacting safety performance from the outset.

According to the tier structure in Figure 2, levels L1 and L2 are categorized as superficial factors. Levels L3 through L5 represent intermediate risk contributors. Levels L6 and L7 comprise the foundational layer of risk, indicating the most deep-seated origins. To ensure the accuracy of the results, the intersection of each of the three layers in the cause-first topology and the result-first topology is identified as the final result under the final two-way verification. The specific safety risk elements assigned to each hierarchical level are identified as follows:

1. Superficial factors: Overweight vehicle passage, inadequate inspection and maintenance, deliberate human sabotage, geological and meteorological hazards prone, poor vehicle safety management, unsound emergency management.
2. Medial factors: Frequent traffic collisions, complex traffic flow, inadequate bridge technical grade, high risk dangerous goods transportation, long bridge service life, unreasonable planning and design.
3. Bottomed factors: Insufficient informatization management, and unqualified management competence. While only a few factors are directly connected, their indirect impact is widespread.

This hierarchical mapping addresses *RO*3 by systematically structuring the risk factors into superficial, medial, and bottomed levels, thereby clarifying the primary pathways through which safety risks propagate during bridge operations.

## 5 Discussion

By integrating empirical data from 132 bridge accident cases with advanced analytical methods, this research provides a comprehensive understanding of multi-level safety risks in bridge operations.

### 5.1 Key findings

Drawing upon 132 documented accident cases, this study identified and analyzed 19 key risk factors influencing the operational safety of bridges by integrating text mining techniques, the association rule algorithm, and the DEMATEL-AISM framework. To ensure the robustness and credibility of the findings, five domain experts, who were previously involved in the refinement and definition of these safety risks, independently reviewed the analysis process and outcomes.

The findings indicate that safety incidents arise from intricate interactions among risk factors, organized into a multi-level hierarchy of superficial, medial, and bottomed levels, rather than from isolated risks or mere hazard accumulation (29). This hierarchical structure provides a clearer understanding of risk transmission mechanisms compared to conventional models such as “human-equipment-environment-management” (70), HFACS (25), and 4M1E (33), which often fail to depict risk propagation over time. Specifically, overweight vehicle passage is identified as the most frequent and significant risk factor, followed by inadequate inspection and maintenance, aligning with findings from Fiorillo and Ghosn (57) and Lou et al. (58). The AISM model highlights insufficient informatization management as a fundamental cause undermining management competence in bridge operations, underscoring that management-related deficiencies form the structural basis for risk proliferation across technical and operational domains. Additionally, unsound emergency management is a critical risk factor, particularly for unpredictable geological and meteorological hazards, which significantly disrupt bridge safety and operational continuity (59).

These findings align with Xiong et al. (72), who used interpretive structural modeling to confirm safety management systems as root causes and surface-level factors like heavy vehicle traffic and inspection activities as immediate contributors to expressway bridge safety risks. Similarly, Andrić and Lu (2) identified natural and geological threats, traffic-related risks, human-induced hazards, and design deficiencies as primary contributors to bridge safety accidents using fuzzy analytical hierarchy processes and fuzzy logic techniques. However, their methodology overlooks managerial factors and causal chains, whereas this study advances the analysis by uncovering hierarchical interactions and highlighting management system weaknesses as fundamental drivers. In contrast to Wang et al. (25), who focused on construction-phase risks and emphasized cognitive factors and safety training deficiencies, this study prioritizes insufficient informatization management and inadequate managerial competence as bottom-level causes in the operational phase, leveraging a data-driven approach that integrates textual data from 132 accident reports with expert assessments.

### 5.2 Theoretical contributions

This study differs from previous research in several important aspects. First, while most existing works have concentrated on construction-phase risks or adopted knowledge-driven assessments, we focus on the operational phase of bridges, a stage often overlooked but with high real-world significance. Second, unlike conventional ISM-based approaches that are constrained by single-directional logic, our use of AISM combined with DEMATEL and Apriori introduces a methodological novelty that enables dual-hierarchy extraction, clearer causal interpretation, and reduced subjectivity. Third, in contrast to machine learning studies that primarily predict isolated structural behaviors (e.g., pavement cracks, ground consolidation, anchor pullout capacity), our approach emphasizes hierarchical causal propagation across 19 operational risk factors, offering systemic insights into how management, environmental, and technical deficiencies interact and escalate into accidents. These contributions collectively advance the methodological toolkit for infrastructure risk analysis and provide a more comprehensive understanding of operational safety risks in bridges.

Furthermore, this study advances the theoretical understanding of safety risk dynamics in bridge operations by developing a multi-tiered hierarchical model that elucidates the complex interactions and propagation of 19 identified risk factors across superficial, medial, and bottomed levels. Unlike conventional frameworks such as “human-equipment-environment-management” (70), HFACS (25), and 4M1E (33), which provide broad categorizations but lack clarity in depicting temporal and causal propagation, this research offers a dynamic and structured representation of risk transmission mechanisms. By integrating text mining, association rule mining, and the DEMATEL-AISM framework, the study addresses critical gaps in causal chain analysis, particularly in the operational phase of infrastructure projects, thereby contributing to the literature on infrastructure safety risk assessment.

Methodologically, this study contributes novelty by employing AISM rather than conventional ISM. While ISM has been widely applied to model hierarchical relations, its single-directional logic often limits the ability to capture antagonistic or bidirectional influences. AISM addresses this limitation by constructing dual simplified topologies from opposing rules, which not only increases model robustness but also provides a richer representation of risk propagation pathways. This methodological enhancement ensures that the causal hierarchies identified in this study are more consistent with the complexity of real-world bridge operation risks.

The emphasis on insufficient informatization management as a fundamental driver extends prior work by Li et al. (34), who identified weak digital integration as a constraint on real-time SHM and risk prediction. This study further refines the role of managerial factors by positioning them as root causes within the safety risk structure, contrasting with Andrić and Lu (2), who overlooked managerial influences in their fuzzy logic-based multi-hazard risk evaluation. Compared to Wang et al. (25), who focused on construction-phase risks and emphasized cognitive factors and safety training deficiencies using DEMATEL-ISM, this research highlights insufficient informatization management and inadequate managerial competence as bottom-level causes in the operational phase. The integration of textual data from 132 accident reports with expert assessments enhances the objectivity and robustness of the analysis, overcoming the subjective scoring limitations of prior studies.

### 5.3 Practical implications

Building upon the causal analysis presented in the previous section, the following section outlines specific management strategies for improving safety risk governance in bridge operation contexts.

#### 5.3.1 Establish an integrated and informatized safety management system

According to results of the AISM model, insufficient informatization management emerges as the fundamental cause, which further undermines management competence in bridge operations. This result underscores that management-related deficiencies form the structural basis for risk proliferation across technical and operational domains. Similar findings have been reported in infrastructure safety literature. For instance, Li et al. (34) demonstrated that weak digital integration constrains real-time SHM and risk prediction in infrastructure systems. Addressing managerial and digital integration shortcomings is essential for breaking the causal chain that drives risk escalation across technical and operational domains. Strengthening BMS through the integration of IoT sensors, BIM platforms, SHM technology, and AI-driven analytics enhances real-time monitoring and predictive maintenance, enabling effective human–machine collaboration (1, 19, 71). This digital transformation not only addresses technical deficiencies but also empowers managers with data-driven insights, thereby improving decision-making capacity and management quality (22). Previous studies confirm that advancing BMS informatization is pivotal for upgrading managerial competencies and ensuring resilient safety governance (1). Therefore, fostering the digital foundation of BMS while simultaneously developing managerial capabilities will provide an effective pathway to mitigate operational risks and improve the overall safety performance of bridge management.

#### 5.3.2 Strengthen vehicle safety management and inspection protocols

The study identifies overweight vehicle passage as the most frequent and significant safety risk factor, followed by inadequate inspection and maintenance. Overloaded trucks are repeatedly documented as causing cumulative fatigue damage and reducing bridge service life, which aligns with the findings of Fiorillo and Ghosn (57) and Lou et al. (58). In practice, vehicle safety management can be improved by deploying weigh-in-motion (WIM) systems and integrating real-time monitoring, which effectively control axle loads and mitigate excessive structural stress (24). On the other hand, inspection and maintenance of bridges can be enhanced through the application of digital twin models and intelligent transportation systems (ITS), which have shown significant potential for improving early detection of defects and optimizing maintenance cycles (60). Moreover, policy frameworks combining enforcement and incentive schemes are recommended to strengthen compliance and promote collaborative governance among freight operators and infrastructure managers (61).

#### 5.3.3 Enhance emergency management for geological and meteorological hazards

Inadequate emergency management has emerged as a critical safety risk factor in bridge operations, especially in the context of geological and meteorological hazards that are often sudden and difficult to predict. These hazards, such as landslides, floods, and strong winds, have been shown to significantly disrupt bridge safety and operational continuity (59). Poor emergency preparedness can exacerbate the impact of such events, leading to greater losses and severe consequences. To address this, robust emergency management systems are essential for timely response and effective risk mitigation. Integrating real-time monitoring tools, such as weather radars and seismic sensors, with early warning systems enables proactive hazard detection and response (62). Additionally, bridges with cultural heritage value (F14) require specialized emergency plans due to their irreplaceable historical significance alongside their transportation functions (63, 64). Strengthening emergency governance while considering the unique vulnerabilities of such bridges provides an effective pathway to mitigate operational risks and enhance overall bridge resilience.

### 5.4 Strengths and limitations

This study contributes several notable strengths. First, by integrating text mining with association rule mining and the DEMATEL–AISM framework, it reduces the subjectivity that often characterizes expert-driven approaches and provides a reproducible and data-driven procedure for risk identification. Compared with traditional ISM models, which have been widely applied in construction safety analysis (29), the AISM method employed here generates both cause-oriented and result-oriented hierarchies, thereby enhancing interpretability and enabling clearer identification of dominant propagation pathways. Second, the use of 132 standardized official accident reports ensures that the findings are representative and traceable, whereas many international studies rely on limited case studies or small expert panels (2, 9). Third, the dual-hierarchy causal structure not only advances methodological rigor but also provides practical insights by distinguishing root managerial deficiencies from surface-level hazards, which is highly relevant for operational safety governance.

Despite these contributions, several limitations warrant further exploration. Firstly, due to the computational complexity of the influence matrix, the scope of analysis was restricted to 19 risk factors. Secondly, the number of expert participants in the evaluation process was relatively small, potentially introducing subjective bias. Additionally, the entire dataset originated from China, which means cultural, social, and economic contexts specific to the region may have influenced the outcomes. Lastly, to maximize the potential of text mining, a standardized accident report format should be developed to ensure consistent data quality.

Future research could address these limitations by expanding the dataset to international accident reports, incorporating multimodal data such as sensor monitoring and inspection records, and combining DEMATEL–AISM with advanced machine learning techniques to enhance scalability and predictive capability.

## 6 Conclusion

The present study extracted safety-related risk elements from 132 documented accident reports using a text mining methodology. After an initial screening and refinement process, 19 key risk factors were identified. Leveraging association rule mining, a dataset was developed that captured 31 significant inter-factor relationships. The DEMATEL method was subsequently applied to determine the relative influence of each factor, while the AISM technique facilitated the construction of a multi-level hierarchical structure. As a result, the risk factors were categorized into seven levels, forming 20 distinct paths of risk transmission.

The findings indicate that factors including overweight vehicle passage, inadequate inspection and maintenance, geological and meteorological hazards, and unsound emergency management are immediate, or direct, contributors to safety incidents during bridge operations. In contrast, insufficient informatization management and unqualified management competence constitute the root causes, shaping and amplifying the influence of other risk factors. These underlying deficiencies form critical pathways for risk propagation and escalation. This research not only clarifies the interaction mechanisms among key risk elements but also offers theoretical guidance for enhancing safety governance in the field of bridge operation and maintenance. A structured understanding of these risk pathways supports targeted interventions at multiple levels—enterprise management systems, technical inspection protocols, environmental hazard resilience, emergency response capacity, and overall risk mitigation strategies. Collectively, these improvements contribute to more effective risk prevention, minimization of operational disruptions, and the establishment of higher safety standards for the national bridge management sector.

## Data Availability

The raw data supporting the conclusions of this article will be made available by the authors, without undue reservation.

## Footnotes

^1^ *https://www.mem.gov.cn*

^2^ *https://www.safehoo.com*

^3^ *http://www.cnbridge.cn*
